# Air purifiers in classrooms for infection control: a pilot study including a randomized multiple crossover trial to evaluate effectiveness in reducing particulate matter

**DOI:** 10.1186/s13063-026-09839-5

**Published:** 2026-07-11

**Authors:** Cathinka Halle Julin, Ingeborg Hess Elgersma, Runar Barstad Solberg, Christine Holst, Arnfinn Helleve, Christopher James Rose, Unni Gopinathan, Atle Fretheim, Petter Elstrøm, Sverre B. Holøs

**Affiliations:** 1 Centre for Epidemic Interventions Research (CEIR), Norwegian Institute of Public Health, Oslo, Norway; 2 Faculty of Health Sciences, Oslo Metropolitan University, Oslo, Norway; 3 Centre for Evaluation of Public Health Measures, Norwegian Institute of Public Health, Oslo, Norway; 4 Cluster for Reviews and Health Technology Assessments, Norwegian Institute of Public Health, Oslo, Norway; 5 SINTEF, Trondheim, Norway

**Keywords:** Air purifiers, Schools, Infection control, Pilot study, Feasibility, Acceptability, Transmission, Air quality, Particulate matter, Absenteeism

## Abstract

**Background:**

Air purifiers with high-efficiency particulate air (HEPA) filters aim to reduce particulate matter from the air, including infectious aerosols, potentially lowering airborne disease transmission in classrooms. This pilot study, informing a larger randomized trial (RCT), explored the acceptability and feasibility of air purifiers in classrooms, and estimated their effect on particulate matter (PM_2.5_) concentration.

**Methods:**

This study, conducted in schools in the greater Oslo area, Norway, from January to April 2023, consisted of two parts lasting 6 and 9 weeks, respectively. In part 1, eligible primary school classrooms received either ceiling-mounted or portable purifiers. In part 2, we conducted a three-arm randomized multiple crossover trial of ceiling-mounted versus portable purifiers versus no air purification. Schools with three classrooms on the same floor with adequate space for air purifiers were eligible. We evaluated acceptability and feasibility of installing and operating air purifiers by collecting interview data from students (part 1) and teachers (parts 1 and 2). The feasibility of collecting consent and questionnaires from students’ legal guardians and student absences from municipality records was explored. We evaluated superiority of air purification versus no air purification and noninferiority of portable versus ceiling-mounted air purifiers on PM_2.5_ concentration using sensor measured air quality data.

**Results:**

Five primary schools (five classrooms) and one adult education school (three classrooms) participated in parts 1 and 2 of the study, respectively. Ceiling-mounted and portable air purifiers were feasible to install and operate and were well-accepted by students (*n* = 11) and teachers (*n* = 7). No harms were reported. Consent and questionnaires were collected from 23 of 114 students’ legal guardians. Collecting absence data from municipality records was feasible, although inconsistencies in teachers’ recording affected weekly accuracy. Air purification was superior to no air purification in reducing PM_2.5_ concentration (*p* = 0.011), and portable purifiers were noninferior to ceiling-mounted units (*p* < 0.001) and significantly reduced PM_2.5_ compared to no air purification (rate ratio 0.71; 95% CI 0.57–0.89).

**Conclusions:**

Installing and operating air purifiers in classrooms is feasible and acceptable. Portable purifiers are user-friendly and reduce PM_2.5_ concentration. It was challenging to obtain consent from students’ legal guardians to collect absence data; municipality records proved more practical. A larger RCT on air purifier effectiveness in schools will be feasible if data collection challenges can be overcome.

**Trial registration:**

NCT06374316 (retrospectively registered 15 April 2024)

**Supplementary Information:**

The online version contains supplementary material available at https://doi.org/10.1186/s13063-026-09839-5.

## Background

During the COVID-19 pandemic, many authorities closed schools worldwide to protect students and teachers. However, closures led to learning loss, mental health issues, and family stress [1–3], highlighting the need for alternative infection control measures. Improved ventilation and air cleaning have been suggested as promising interventions to avoid school closures in future pandemics [4].

Recent studies in classrooms and other indoor settings have indicated that air purifiers with High Efficiency Particulate Air (HEPA) filters may reduce the concentration of airborne particles, including those carrying SARS-CoV-2 virus [5–11]. Modeling studies further support the potential effectiveness of air purifiers in reducing infection risk [12, 13]. While these studies suggest that air purifiers can be a valuable tool in mitigating the risk of infectious aerosols being transmitted, research on the real-world effect of air purifiers remains limited. In observational studies, factors like existing ventilation, classroom size, and local conditions can confound the observed effect of air purifiers, underscoring the need for randomized controlled trials (RCTs) to better understand their impact on disease transmission [14].

To date, three randomized trials investigating the impact of air purifiers in school settings are ongoing or have been completed. Two of these trials—the Class-ACT study in the UK (ISRCTN46750688) [15], and a trial in Italy—have been completed, but the findings from the UK study have yet to be published. The Italian study, published in a working paper, reports that the intervention reduced school absenteeism by 0.7 percentage points, corresponding to a 12.5% relative reduction. The effect estimate is significant at the 10% level (*p* < 0.1), authors noting that their study may be underpowered to detect significant effects on student absence. A third trial, in Massachusetts, USA (NCT05953233), is still underway. More randomized trials will be needed to establish a robust evidence base for the real-world effect of air purifiers in schools on disease transmission.

Before conducting a large trial, it is important to assess whether installing and operating air purifiers in classrooms is feasible, and if teachers and students find the devices acceptable. It is also important to determine whether air purifiers have a measurable effect on the concentration of airborne microbes, and whether there is a difference between types of air purifiers (ceiling-mounted versus portable air purifiers). Direct measurements of concentration of bacteria and viruses in the air via viral air samples can be considered costly and time consuming, whereas low-cost optical sensors could monitor total particle matter (PM) concentration at higher time-resolution with limited effort. Optical sensors typically detect PM > 0.3 µm. Studies have suggested that droplet nuclei of approximately 1 µm diameter are considered highly relevant for airborne transmission of viruses [12]. Thus, a reduction in PMs in the size range 0.3–2.5 µm (PM_2.5_) is a reasonable indicator for reduction of infectious aerosols. Volatile organic compounds (VOC) may influence perceived indoor air quality (PAQ) in schools. Air filters may affect VOC concentration and perceived air quality by primary or secondary emissions from materials deposited in filters [16, 17], or by adsorption by adsorbents incorporated in some filters. Changes in PAQ due to air purification may affect user acceptability as well as user habits with possible impact on infectious aerosols, such as how often classrooms are aired. Electronic VOC sensors are an affordable alternative for detecting changes in VOC concentration, but with some known limitations [18–20].

The main objectives of this pilot study are as follows: (1) to assess the acceptability and feasibility of a large-scale randomized trial in Norwegian schools which includes installing and operating ceiling-mounted and portable air purifiers; (2) to determine whether air purification is superior to no air purification in lowering the amount of particulate matter (PM_2.5_) in classrooms; and (3) to assess whether portable air purifiers are non-inferior to ceiling-mounted air purifiers in lowering the amount of particulate matter (PM_2.5_) in classrooms.

Together, the findings from this pilot study informed the design of a large-scale randomized trial in Norwegian schools aimed at evaluating the effect of air purification on disease transmission [21].

## Methods

We understand a pilot study to be “a study in which a future study or part of a future study, is conducted on a smaller scale to ask the question whether something can be done, should we proceed with it, and if so, how” [22]. Assessing feasibility is an inherent part of a pilot study and acceptability a prerequisite for feasibility. Acceptability ascertains whether the participants receiving the intervention consider it to be appropriate [23]. We tested our intervention on a small scale, gathered data on the feasibility and acceptability of the intervention (installing and operating air purifiers), and we explored approaches for recruitment of study participants and data collection.

An assessment of feasibility in this study arguably includes determining whether air purifiers produce a measurable reduction in particulate matter representing infectious particles in classrooms. A change in exposure is a prerequisite for proceeding to a larger trial on the effect on disease transmission; without such a change, no effect on the outcome can be expected. For clarity in reporting this pilot study, however, we have separated the objectives that concern the effect of air purifiers on particulate matter (objectives 2 and 3) from the overall assessment of feasibility and acceptability (objective 1).

The methods section is structured as follows: We first describe the study design and setting, as well as participants and recruitment, followed by a description of the intervention. We then present the data collection and analysis separately for acceptability and feasibility (objective 1) and for the effectiveness of the air purifiers in reducing PM_2.5_ (objectives 2 and 3).

### Study design and setting

This pilot study was conducted in Eastern Norway and consisted of two parts. In part 1, either ceiling-mounted or portable air purifiers were installed in primary school classrooms. In part 2, a three-arm randomized multiple crossover design was used to assess the effect of ceiling-mounted and portable air purifiers versus no air purification on air quality in classrooms. Data from both part 1 and part 2 were collected to address feasibility and acceptability (objective 1). Data from part 2 informed objectives 2 and 3. The study design is depicted in Table 1***.***

**Table 1:**
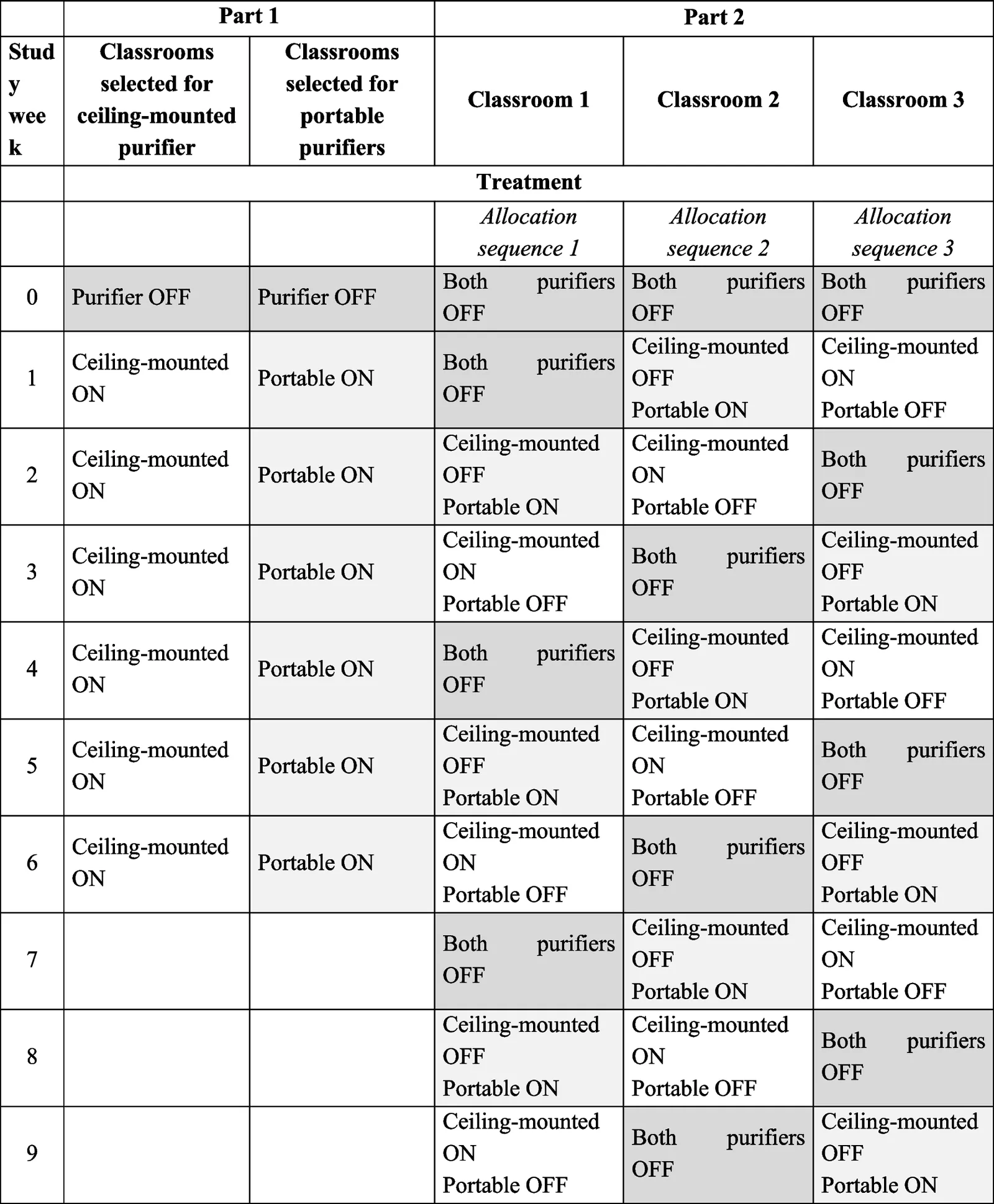
Study design—weekly treatment schedule for the use of the air purifiers in the various classrooms in part 1 and part 2 of the study, according to study week

In part 1, classrooms were assigned to either one ceiling-mounted or two portable air purifiers. We aimed to equip approximately half of the classrooms with one of the two air purifier types, considering the available floor space and classroom infrastructure. During the 6-week study period (study weeks 1–6), the air purifiers were operational every day during school hours, except Tuesdays (see Protocol Deviations).

In part 2, each of the three classrooms was equipped with a ceiling-mounted purifier, a portable air purifier, and two air quality sensors. Classrooms were randomized to use ceiling-mounted, portable, or no air purifiers during school hours per the multiple crossover schedule shown in Table 1. We assigned a label to each classroom and used a computer-based randomization method to allocate each classroom to one of the predetermined schedules.

The purifiers were not operated on the weekends (washout periods) to reduce carryover effects. The weekends were considered sufficiently long washout periods as participating classrooms were mechanically ventilated at such a rate that most of the air in the room (the potential carryover effect) would have been changed within a few hours.

In both part 1 and part 2, air quality sensors were installed in all classrooms. Measurements began from study week 0, to establish baseline air quality. The sensors monitored air quality throughout the study period at 5–10-min intervals. Teachers were allowed to air out the classrooms according to their usual needs during the study period.

The study was registered at clinicaltrials.gov (NCT2302888), and the protocol was published at Zenodo [24]. We followed the CONSORT 2010 checklist of information to include when reporting a pilot or feasibility trial [25] (see supplementary material, Table 1).

### Participants and recruitment

For part 1, all primary schools in Eastern Norway were eligible to participate. The schools were asked to nominate one classroom in the 5th to 7th grade (i.e., children aged 10–12 years), with a minimum of 15 students. Children in these grades were eligible because they leave the school area when school is finished and because schools are likely to record absences more consistently for 7th-grade students (the final year of primary education). Children in the 1 st to 4th grades (i.e., children aged 6–9 years) were ineligible because they can participate in after-school programs that use rooms other than students’ regular classrooms: this could result in spillover effects in a future RCT on the effect of air purifiers on disease transmission if the unit of randomization was class. Classrooms with sufficient space to accommodate at least two portable air purifiers and which had the necessary infrastructure to install a ceiling-mounted air purifier were eligible.

For part 2, all schools in the greater Oslo area in Norway were eligible to participate if they had at least three similar classrooms on the same floor, sufficient floor space to accommodate at least two portable air purifiers, and the necessary infrastructure to install a ceiling-mounted air purifier. The three nominated classrooms needed also to be comparable in terms of usage and be rated by principals and/or teacher as having moderate or poor air quality. The eligibility criteria differed between part 1 and part 2 because part 1 focused on collecting acceptability data from a single classroom of 5th to 7th graders, whereas part 2 required three adjacent classrooms to enable comparisons across classrooms. While part 2 was less restrictive with respect to student grade, it imposed stricter classroom requirements, reflecting the different objectives and study design for each part.

Each school principal was responsible for deciding to accept the offer to install air purifiers and air quality sensors for the purpose of this study. All class teachers and primary school students in the participating classrooms were eligible to take part in interviews and answer weekly questionnaires if written informed consent had been given. Information letters and consent forms were sent directly by email to the class teachers, and to the legal guardians through the school’s message system, with at least one reminder. In some schools, printed copies were also distributed to the students. Written informed consent was acquired from all legal guardians of students who participated in interviews and/or answered questionnaires.

### Interventions

We used a ceiling-mounted air purifier (Euromate Visionair Blue Line 2, Euromate, 4817 ZD Breda, the Netherlands) and a portable air purifier (Mill Silent Pro Air Purifier, Mill International AS, 1361 Østerås, Norway) in this study. Both were supplied with HEPA filters with activated carbon as a secondary filtration step. Manufacturers’ specifications and introductory test results are listed in supplementary materials, Table 2. We selected these purifiers through a limited tender process where suppliers were asked to suggest products suitable for a 60 m2 classroom that could be operated within the noise level limits described below. We did not replace the HEPA filters during the study period. The manufacturer of the portable air purifiers recommended replacing their HEPA filters after approximately 12 months of normal use, whereas the supplier of the ceiling-mounted air purifiers specified in the tender documents that their filters would be replaced every 3 months. However, the actual replacement interval varies in different settings depending on factors such as dust load and fan speed [26].

Classrooms in part 1 allocated to ceiling-mounted purifiers had a single device mounted in the center of the room, while classrooms with portable air purifiers had two devices each. Part 2 classrooms received both purifier types. The portable purifiers were positioned, if possible, at least one meter from any wall, at least one meter from the closest seat, and on opposite sides of the room. Placement decisions were made through discussions between researchers and teachers, considering room configuration and use. Measures were also taken to minimize the risk of tripping over power cords and purifier units.

The initial intensity level of the air purifiers was set at an assumed acceptable level based on the Norwegian standard for technical installations in schools (< 35 dB(A)) [27]. Portable units were set to level 4 (of 8), while ceiling-mounted units were programmed by the supplier to level 3 (of 8). In selected schools, portable purifiers operated at level 5 (of 8) for 1 week after the 6-week study period, to evaluate acceptability by students and teachers.

### Acceptability and feasibility

We identified the following aspects as important to explore in relation to whether the pilot could be scaled up to a full-size randomized trial:

- Feasible and acceptable data collection procedures: Our outcome in a larger trial would be a measure of disease transmission, and we explored the feasibility of collecting data for four potential outcome measures: self-reported absences in students and/or teachers, self-reported respiratory infections in students and/or teachers, administratively recorded absences, and lab-confirmed respiratory tract infections obtained through testing.
- Acceptability of intervention: whether the presence of ceiling-mounted or portable air purifiers in the classroom was acceptable to students and teachers.
- Feasibility of installing and running air purifiers: whether the air purifiers could be installed and operated smoothly.

#### Data collection

To evaluate the feasibility of collecting outcome data directly from students and teachers, we tested out acquiring consent and collecting weekly questionnaires on absences from teachers and legal guardians of students. The weekly questionnaire was subsequently sent via SMS to the consenting legal guardians and teachers. The questionnaires collected information about absences (number of days per week) and reasons for absence (airway infection, or other newly developed illness, or health problems not caused by newly developed illness, or other reasons). Teachers were additionally asked about air quality using questions from the MM040 survey [28] in the baseline and end questionnaires to assess the feasibility of collecting such data.

To explore the collection of anonymous absence data at class level, the principals were consulted to identify the most practical approach. Consequently, the absence data were obtained directly from the municipality records rather than from the individual schools. As these data were aggregated at the group (class) level and did not include any personally identifiable information, informed consent for these specific data was not required.

We conducted qualitative group and individual interviews with both teachers and primary school students, to examine acceptability of having air purifiers running in the classrooms, as well as willingness to participate in a larger study to evaluate the effect of air purifiers, which included exploring the acceptability of different outcome data collection procedures, including testing for respiratory tract infections. All interviews with teachers were conducted online via Microsoft Teams. Each was moderated by one researcher, while one or two others observed, took notes, and occasionally asked follow-up questions. Three of the interviews were recorded, and all followed an informal conversational style guided by a semi-structured interview guide (see supplementary material, teachers interview guide).

Group interviews with primary school students were conducted face-to-face after school. One researcher provided an overview of the study objectives and explained the interview procedure, while another took notes and posed follow-up questions. A semi-structured interview guide was followed (see supplementary material, students interview guide). The sessions were voice recorded. Each group consisted of 5–7 students, and they were offered pizza for their participation.

All group and individual interviews with teachers and students lasted between 30 and 60 min. Afterwards, the researchers conducted a debrief session.

The feasibility of turning the portable air purifiers on and off daily, and monitoring if they operated as planned, was explored using various approaches. At all six schools, we tested a Weekly Electronic Timer (Emos TS-ED1, Elkjøp, Oslo, Norway) that automatically activated the portable devices at 8:00 AM and deactivated them at 16:00. We also tested controlling the portable purifier through the Mill app (Mill International AS, Østerås, Norway) at one school and by using a Bluetooth smart plug (Plejd SPR-01 Smart Plug, Plejd AB, Mölndal, Sweden) at another school. These observations were documented in field notes. The ceiling-mounted purifiers were pre-programmed by the supplier to follow our predefined schedules, so alternative control methods were not explored.

#### Qualitative data analysis

Qualitative data were descriptively analyzed [29] using a modified content analysis approach [30]. Predefined themes related to feasibility and acceptability guided the analysis. One researcher reviewed the recordings and transcripts, noting findings, codes and quotes related to themes. Key highlights were documented separately. To ensure consistency and credibility, while maintaining a descriptive focus rather than engaging in deep interpretation, three researchers collectively reviewed and discussed the findings from each interview. A comprehensive summary document was subsequently established. Once all findings had been summarized, two researchers who had not taken part in interviews reviewed findings from the qualitative material and how these results were reported in the manuscript.

By employing method triangulation, which involved comparing field notes, data from qualitative interviews and survey data, we assessed acceptability and feasibility.

### Effectiveness of air purifiers in reducing PM_2.5_

#### Data collection

Air quality was monitored by one Airthings Space Pro (Airthings AS, 0167 Oslo, Norway) and one IOT Air Quality Sensor (Digiref AS, 3117 Tønsberg, Norway), mounted on walls in the classrooms. Both sensors recorded air temperature, air relative humidity and CO_2_ concentration at 5-minute intervals. Fine particles were reported as mass concentration of PM_1_ and PM_2.5_ at 10-min intervals (Airthings) or as mass concentration, number concentration, and typical particle size of PM_2.5_ at 5-minute intervals (Digiref).

#### Statistical analyses and sample size calculations

The power calculation for objectives 2 and 3 was based on a one-sided test of noninferiority of portable versus ceiling-mounted air purifiers; the noninferiority hypothesis is described in detail in section 8.4 of the statistical analysis plan [31]. We estimated that at least 28 outcome measurements would be required in each group. Accordingly, we planned to run part 2 for 6 weeks and measure outcomes at least daily during weekdays when the classrooms were in use. In practice, the outcomes were measured more frequently (see Air quality in Methods).

Outcome data for part 2 were limited to teaching hours (8:30–15:00). We included data from breaks between teaching sessions because students were allowed to stay in the classroom during breaks.

All statistical analyses were performed as prespecified [31], except as noted, using Stata 18 (StataCorp LLC, College Station, Texas, USA). We analyzed the effect of air purification on the primary outcome PM_2.5_ and on the secondary outcome VOC. We modeled outcomes as counts using negative binomial regression, which was selected over zero-inflated negative binomial and Poisson models for intention-to-treat (ITT) analyses of both outcomes. Model selection was performed using the Akaike information criterion as prespecified. We therefore also used the selected model for the per-protocol (PP) analyses, which excluded measurements made when the air purifiers were not running as planned. All analyses accounted for the crossover design, sensor type, first-order autocorrelation (lagged outcomes), and clustering within classroom via cluster-robust standard errors. We treated undefined lags (e.g., the first measurement of the day, for which no preceding value is available) as missing at random (MAR) under the assumption they would be highly correlated with outcome and the constant term. We generated an indicator variable to identify each undefined lag and estimated the missing values using full information maximum pseudolikelihood [32]. Exposure was defined as the time between successive outcome measurements. Treatment effect was estimated as rate ratio (RR), where RR > 1 favors no air purification.

We used Stata’s xpopoisson command to perform cross-fit partialling-out lasso Poisson regression [33] in a prespecified exploratory analysis to identify time-varying covariates that may be associated with the primary outcome and assess the sensitivity of the treatment effect estimates to these covariates. The lasso penalty was chosen using 10-fold cross-validation to select among the following covariates, which were then partialled out: indoor relative humidity; the school’s existing ventilation system setting (low or high); weekday; baseline morning PM_2.5_ (modeled on the log scale); number of students attending class; and mean outdoor temperature (data downloaded from [34]). We had planned to include indoor temperature and indicators of undefined lagged PM_2.5_ but had to omit these due to nonconvergence. We had also planned to include indoor CO_2_ but had to omit this because one of the sensors did not measure CO_2_ in one of the classrooms due to a defect.

Finally, we performed a two-sided joint test of the hypothesis of no effect of any versus no air purification (reported as a test of superiority) and a one-sided noninferiority test [35] of portable versus ceiling-mounted air purifiers using the prespecified noninferiority margin RR = 1.4. The margin was chosen to preserve at least 80% of the treatment effect for PM_2.5_ reported by Han et al. [36]. We report two-sided 95% confidence intervals and use the conventional *p* < 0.05 significance criterion throughout. We used the Cochrane Risk of Bias 2 tool for crossover studies [37] to formally assess the possible weaknesses of part 2.

### Protocol deviations

We decided to deactivate air purifiers in part 1 on Tuesdays to evaluate whether teachers and students could notice if the purifiers were operational, in terms of perceived air quality and noise levels. We extended the study period of part 2 by 3 weeks after a preliminary analysis showed mean classroom particle density was substantially lower than anticipated (1.44 µg/m versus 10 µg/m^3^). This decision was taken after we published the protocol but before we estimated any treatment effects or tested any of the prespecified hypotheses.

### Patient and public involvement

Prior to recruitment of schools, we sought input from a number of municipal Chief Infection Control Officers on study design and recruitment of schools. We also informed and sought comments from representatives from the teachers’ union. After recruitment of the schools but prior to the start of the study we involved school principals. As this was an acceptability and feasibility study, feedback from participants was a crucial part of the data collection. We, however, did not seek any involvement outside of the participating schools of public representatives in the design, implementation or analysis of the study as we assessed our involvement of participants as sufficient.

## Results

### Recruitment

This study took place from the 8th of January until the 12th of April 2024 (see timeline of the study in Table 2), and six schools participated in the study. Five primary schools (A–E) were recruited for part 1 of the study, with one classroom participating at each school. For part 2 of the study, a single school was recruited (school F), providing three classrooms. This was an adult education school, chosen for this part of the study as it was the only recruited school that could provide three comparable classrooms on the same floor. Characteristics of all eight classrooms are detailed in supplementary materials, Table 4. The sensors and air purifiers were placed in part 1 classrooms as described in the methods section. The placements in part 2 classrooms are shown in supplementary materials, Fig. 1.

**Table 2:**
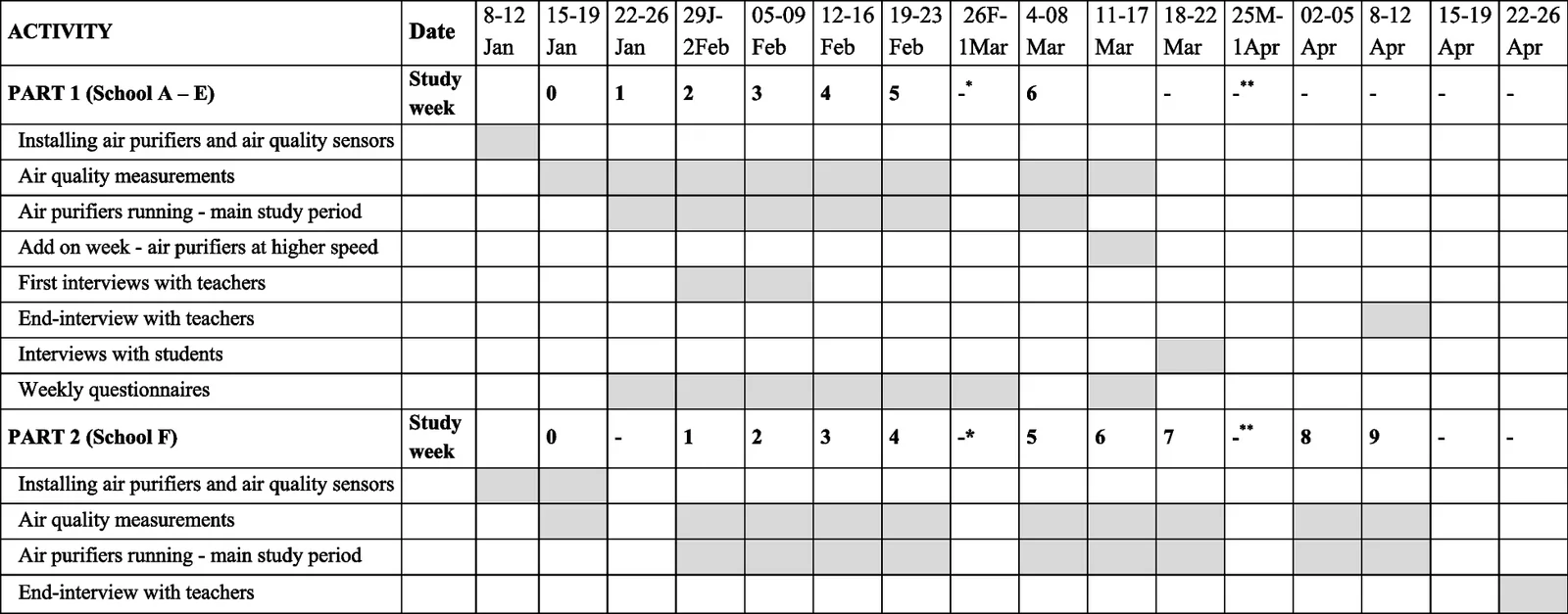
Timeline of the study. Grey color indicates that the activity was performed

^*^Winter Holiday. ^**^Easter Holiday

In part 1 of the study, all five teachers who were approached for interviews and weekly questionnaires consented to participate. Of the three teachers approached in part 2, two consented and took part in interviews. In total, we performed one individual interview and four group interviews with teachers during the study.

In the part 1 schools, a total of 114 students were eligible to receive invitations for interviews and questionnaires through the school’s message systems. However, a communication issue at one school meant that the invitation was not sent to their students as planned. As a result, 94 of the 114 eligible students received the invitation. Out of these, consent was obtained from the legal guardians of 34 students to answer the questionnaires. However, eleven students were excluded due to missing consent from one of their legal guardians, resulting in a final group of 23 (20.2%) students. Of the 23 students, 16 also consented to participate in interviews. We interviewed 11 of these, four from a school with ceiling-mounted purifiers and seven from a school with portable purifiers in two group interviews at the respective schools. In total, we performed one individual interview and four group interviews with teachers during the study.

### Acceptability and feasibility

#### Feasibility of collecting outcome data on absences from teachers and students

All teachers (part 1) consented to take weekly the questionnaire. All teachers responded to all questionnaires.

Out of the 23 students where we received parental consent, 11 completed all questionnaires. The weekly response rate is shown in supplementary materials, Fig. 2. Surveyed parents reported that nine of the 21 absence days (approximately 45%) were due to respiratory tract infections. Three of 21 days were attributed to non-respiratory illnesses, 3 days to chronic diseases, and 7 days to other reasons (Note: The total number exceeds 21 because parents could assign multiple reasons for a single day). With the survey covering a total of 650 student-days (not all legal guardians answered every week), on average, 3.2% of the students were reported as absent on a given day.

In the interviews, the teachers expressed that it could be challenging for the study team to obtain consent from parents via links and SMS in a larger study. They anticipated that this approach could generate a low response rate with a socioeconomic gradient. The teachers preferred to collect consent on behalf of the study team, as it would allow them to track which students had delivered their answers. They also emphasized the need for shorter and simpler language in the information and consent forms provided to parents.

In terms of collecting survey data from teachers, they noted that it was crucial for the questionnaires to be concise for quick submission on-the-go. In the pilot study, the initial and final questionnaires were slightly longer than the intermediate ones, causing the teachers to delay responding and risking non-response.

#### Feasibility of collecting recorded absences

We received aggregated class absence data from the municipality’s administrative system for absence registration. This system relies on teacher registrations. However, the teachers informed us that the frequency of registering absence in this system varied. Potential delays in reporting make weekly data potentially erroneous. In total, there were 285 registered days of absence among 114 students during the 8-week follow-up period. On average, 6.25% of the students were registered as absent on a given day.

#### Acceptability of testing for respiratory tract infections among students and teachers

In the group interviews, we also asked teachers and students what they felt about swab testing for respiratory viruses and reporting the results back to the investigators as part of a future larger study. Both teachers and students responded that this was highly dependent on how frequently such testing would occur. Teachers were mainly positive to do regular testing every week. Students generally noted that they felt that nasal swabs were very uncomfortable. One stated that “I would skip school if I would have to test before each school day.”

Based on the survey responses from 14 respondents, six (42.9%) parents preferred to use a test swab (equal to that of a COVID-19 rapid test) and return the results to the researchers by SMS, while four (28.6%) preferred to use a test swab, send the test to a lab, and receive the result from the lab. The remaining four (28.6%) preferred not to have their child participate in testing for respiratory tract infections.

#### Acceptability of the intervention

In the interviews, none of the teachers in part 1 schools found either type of air purifier bothersome. They had no objections to having air purifiers permanently installed if it would allow schools to remain open during a pandemic. Teachers in classes with portable purifiers felt that while the units occupied space and could limit classroom rearrangement during lessons, the slight inconvenience was worthwhile: “It’s a small thing to have in the classroom to maintain normal school operation.” [same person]: “They’re a bit big and clumsy, but if it can help reduce transmission, enabling a relatively normal daily life in case of a new COVID situation, then it’s not a big sacrifice. They haven’t been a major inconvenience.” One part 2 teacher reported noticing no difference in air quality between weeks with and without air purification, regardless of whether the ceiling-mounted or the portable air purifier was on. The second teacher noticed a positive difference with the air purifiers, stating that “the portables were more pleasant, because they were placed closer to me, but at the same time they occupy more of the floor which makes it more difficult to move around. In sum, there are advantages and disadvantages with both types”.

In the interviews, all students expressed a positive overall experience of having air purifiers in the classroom, despite occasionally noticing sound from both air purifier types and minor physical obstruction from the portable units. All students supported having air purifiers in the classroom throughout the entire school year. There were no noticeable differences between the student’s perceptions of the ceiling-mounted versus the portable purifier, except the obstructive nature of the portable units.

In the interviews, students and teachers indicated that the noise level from the air purifiers was not considered bothersome. Only students sitting close to the purifier reported hearing it: “It sometimes turns on and off; I’ve sat behind it twice, so I kind of felt that it made a bit of noise and there was some air coming from it under the desk, it was kind of nice […] I don’t like it when it turns itself on and off all the time.” When one of the student groups was prompted with whether the sound was like a kitchen fan, they agreed, but also noted that it was “Very difficult to hear it - you can only hear it if you're close and quite quiet.”

Noise measurements taken by the study team in selected classrooms showed that the portable purifiers were well below the recommended 35 dB(A), while the ceiling-mounted were slightly above this threshold, both for the room average and most affected person (see supplementary materials, Table 2).

Regarding changes in air quality after installing the air purifiers, some teachers reported a positive impact, noting fresher air and a reduction in unpleasant odors, while others noticed no difference. Similarly, many students expressed that the air felt fresher and less stuffy, with a noticeable reduction in classroom odors. Some students stated that the air quality before air purifier installation was bad, “like being in space sharing only one tank of air for everyone.” Some students reported fewer headaches and feeling healthier after the introduction of the air purifier into the classroom, while others did not notice any differences.

#### Feasibility of installing and running air purifiers

The setup process for the portable purifiers was found to be straightforward. However, the location and availability of power sockets as well as the classroom layout sometimes required adaptation of the placement. In interviews, the teachers’ expressed willingness to install the purifiers for a study, provided that dedicated time was allocated by the school management in their calendar for the installation. However, detailed instructions must be given to ensure optimal placement of the purifiers, considering factors such as existing ventilation and windows.

The childproofing mechanism on the purifiers effectively prevented students from tampering with settings, ensuring that the devices ran at the planned speed throughout the entire study period. The most common issue was students unknowingly unplugging the purifiers to use the wall outlet for other electronic devices.

During the 6-week study period, there were four instances across the five schools where objects fell or were inserted into the air purifiers. In each case, teachers easily resolved the issue by opening the purifiers and removing the objects with guidance from the study team. The ceiling-mounted purifiers operated as planned throughout the study period, except for a few instances where they were accidentally unplugged from wall power outlets. To prevent this from happening, strong tape was used to mark the plugs to indicate that they should not be unplugged.

Additional findings regarding the implementation and monitoring of air purifiers in classrooms as well as the feasibility of viral monitoring are described in the supplementary materials, iii. Additional findings.

### Effectiveness of air purifiers on particulate matter in classrooms

**Fig. 1:**
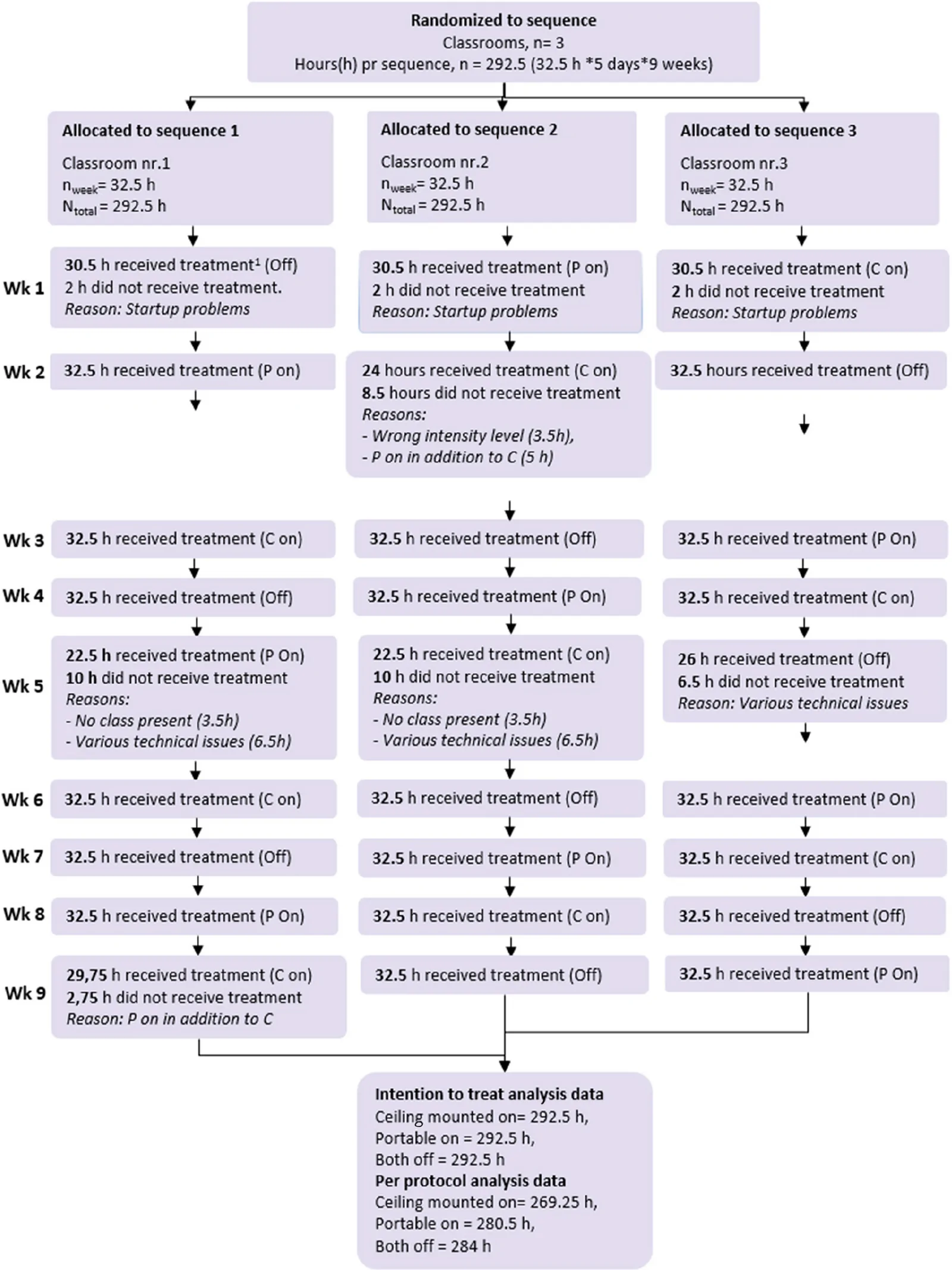
Flow of the randomized classrooms and the resulting “Intention To Treat” and “Per Protocol” analysis data sets. The allocation sequences are described in Table 1. ^1^Throughout the flow chart, “Received treatment” indicates that the study group did not receive any information that the treatment was NOT delivered. Abbreviations: C, ceiling-mounted; P, portable; Off, both purifier types are off

**Fig. 2:**
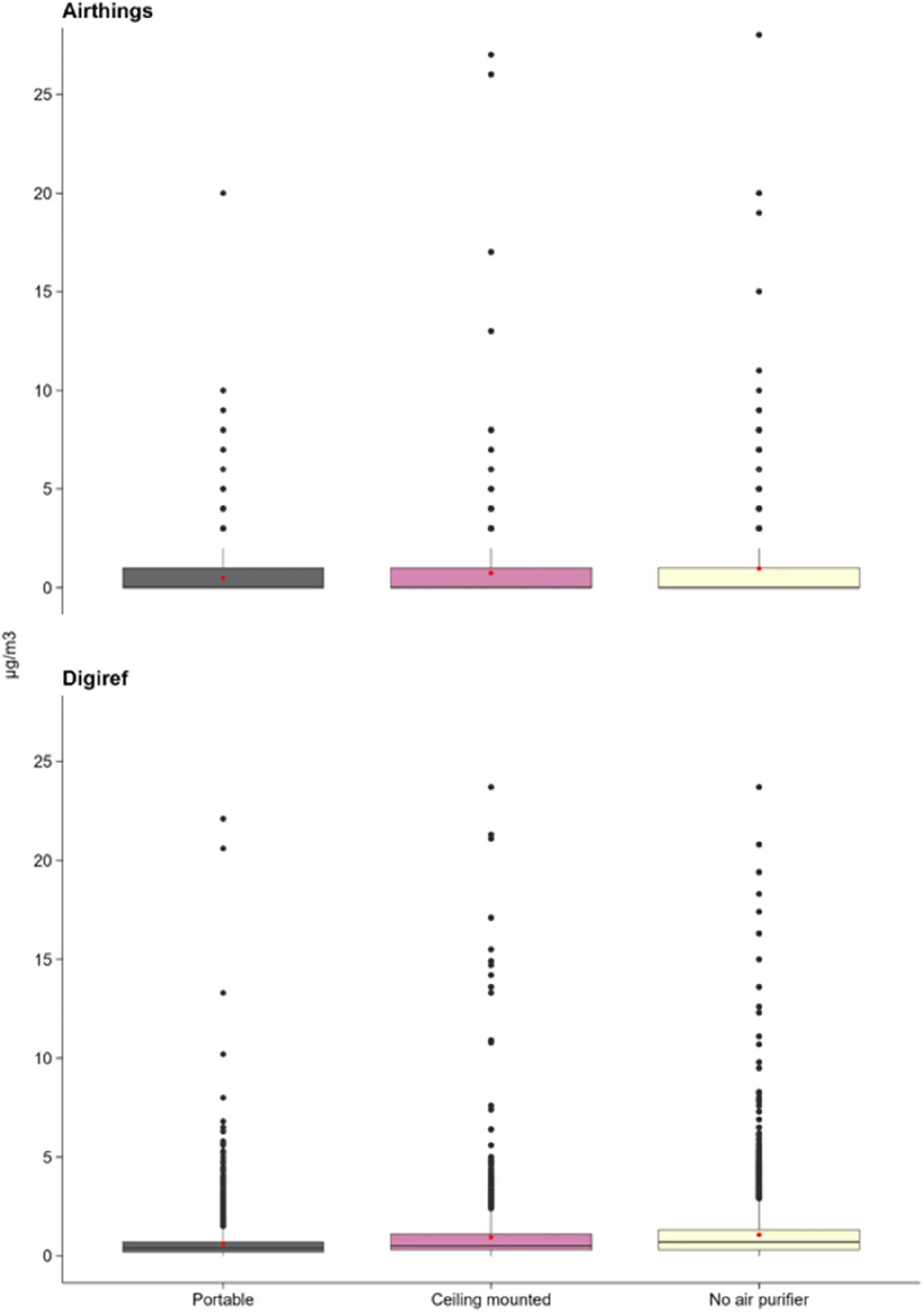
Boxplot of PM_2.5_ count in classrooms from school F (part 2) according to purifier status (portable air purifiers on, ceiling-mounted air purifier, no air purifier on) for each sensor type (Airthings (upper) or Digiref (lower))

In total, we observed air quality for 877.5 h in the part 2 classrooms. The ITT dataset included all measurements, while the PP dataset excluded 43.75 h (see Statistical analyses and sample size calculations and Fig. 1). Distributions of PM_2.5_ in the classrooms during the study period with portable, ceiling-mounted, or no air purifiers are shown in Fig. 2.

The ITT analyses (Table 3) provide evidence against the joint null hypothesis that both portable and ceiling-mounted air purifiers have no effect on PM_2.5_ (*p* = 0.011). The rejection is largely attributable to portable purifiers (RR = 0.71; 95% CI 0.57–0.89), while the estimate for ceiling-mounted purifiers was smaller and imprecise (RR = 0.85; 95% CI 0.65–1.11). Neither type of purifier appears to have an effect on VOC. Portable air purifiers were found to be noninferior to ceiling-mounted air purifiers (*p* < 0.001) because they appear to be superior to no air purification for PM_2.5_ and no effect was observed for either type of purifier for VOC.

**Table 3:** Estimates of treatment effect for the primary and secondary analyses, for the intention-to-treat and per protocol data

	**Type of air purifier**					**Superiority of air purification**^**3**^	**Noninferiority (portable vs ceiling)**^**4**^
	**None**	**Ceiling- mounted**	**Portable**	**Ceiling- mounted**	**Portable**		
	Mean^1^	Mean^1^	Mean^1^	RR^2^(95% CI)	RR^2^(95% CI)	*p*-value	*p*-value
**Intention to treat estimates**							
PM_2.5_	1.03	0.84	0.55	0.85 (0.65–1.11)	0.71 (0.57–0.89)	0.011	< 0.001
VOC	192.39	197.78	202.85	1.05 (0.99–1.12)	1.03 (0.90–1.18)	0.136	< 0.001
**Per protocol**^**5**^ **estimates**							
PM_2.5_	1.03	0.84	0.55	0.86 (0.65–1.14)	0.70 (0.56–0.87)	0.005	< 0.001
VOC	192.39	197.78	202.85	1.07 (0.99–1.15)	1.03 (0.90–1.18)	0.118	< 0.001

*Abbreviations*: PM, particulate matter; VOC, volatile organic compounds

^1^Sample means are unadjusted and do not account for the crossover design

^2^Rate ratios (RRs) are adjusted for the crossover design, sensor type, first-order autocorrelation, and clustering within classroom. RR < 1 favors either ceiling-mounted or portable air purification over the reference (no air purification)

^3^Two-sided *p*-values for the joint null hypothesis${H}_{0}:\mathrm{log}{RR}_{CN}=\mathrm{log}{RR}_{PN}=0$, where${RR}_{CN}$and${RR}_{PN}$are rate ratios comparing ceiling-mounted and portable air purifiers to no air purifiers, respectively

^4^One-sided *p*-values for the null hypothesis${H}_{0}:\mathrm{log}{RR}_{PC}\ge \mathrm{log}m$, where${RR}_{PC}$is a rate ratio comparing portable to ceiling-mounted air purifiers and$\mathrm{log}m$is the prespecified noninferiority margin

^5^The estimation sample was restricted to exclude measurements made when air purifiers were not running as planned

The time-varying covariates selected by the lasso procedure were indoor relative humidity, weekday, baseline morning PM_2.5_, and the school’s existing ventilation system setting (supplementary materials, Table 5). The result of this analysis further supports the superiority of air purification but should be interpreted cautiously because we were unable to account for undefined lags, could not include all time-varying covariates, and it was not possible to use a negative binomial model for this analysis because Stata does not currently offer xpopoisson-like commands for negative binomial models.

Our risk-of-bias assessment for part 2 is described in the supplementary materials, risk-of-bias assessment**.** Our overall assessment of the risk of bias is low, as i.e., the statistical analysis was prespecified, the delivery of the assigned intervention was not prone to be affected by assessors or participants, and there was minimal loss to follow-up.

### Other findings

#### Harms

No unintended consequences or harms connected to the installation or operation of the air purifiers were reported in either for both part 1 or part 2.

#### Air quality in the classrooms

Baseline measurements of air quality during study week 0 in all the participating classrooms are shown in supplementary materials, Table 3. The CO_2_-levels in part 1 (schools A–E) were with few exceptions below the guideline level of 1000 ppm, while School F had median values exceeding this, indicating inadequate ventilation at this school. PM_2.5_ readings were generally low compared to the suggested indoor guideline of 15 µg/m^3^ (daily average) [38]. School D had particularly low readings of PM_2.5_. Median indoor relative humidity was below 20% in schools A–E and only slightly higher in the less ventilated school F. No national guideline exists for indoor relative humidity, but values between 20 and 40% in heated rooms in winter are considered normal. VOCs vary over a wide range in all the classrooms. National guidelines exist only for individual compounds, and not for total VOCs.

To get more insight into the air quality in the primary school classrooms, we further explored the CO_2_, VOC, humidity, and PM_2.5_ during school hours in the 6-week study period, in the classrooms in part 1 (see supplementary materials, Fig. 3). The daily patterns were similar for all schools and showed that CO_2_, VOC, and relative humidity are clearly related to occupancy. PM_2.5_, on the other hand, seemed less related to occupancy, indicating that people are not the dominant source of small particles.

## Discussion

We found that installing and operating air purifiers in classroom settings is both feasible and generally well-accepted by teachers and students. Over the 9-week study period, the air purifiers significantly reduced PM_2.5_ in the classrooms, with portable being non-inferior to ceiling-mounted purifiers. This suggests that air purifiers may improve air-quality in classrooms.

### Comparison to previous studies

Our findings align with the majority of previous studies that have demonstrated that air purifiers are an acceptable and feasible intervention in classrooms and that they may effectively reduce particulate matter [6, 8, 9, 36, 39]. In contrast, one study did not find a reduction in particulate matter by portable air purifiers [40], a discrepancy which may be related to factors such as the placement of purifiers, existing ventilation systems and routines, the chosen intensity levels of the purifiers, or the study design. We did not observe an effect of air purifiers on VOC concentration. This result complements a recent study on the effects of air purifiers in university classrooms which found that for seven out of eight air cleaners, the CADRs for VOCs were significantly lower than the corresponding CADRs for PM [41].

### Relevance of findings for a trial on the effect of air purifiers on disease transmission

Our analyses indicated that indoor relative humidity, weekday, baseline morning PM_2.5_, and the school’s existing ventilation system settings were associated with the concentration of particulate matter. Thus, adjusting for these covariates may be useful in future randomized trials to increase effect estimate precision.

We believe that identifying appropriate intensity levels and exploring how air purifier status can be monitored are the most important aspects when evaluating feasibility of air purification in classrooms. Higher intensities are likely more effective in removing particulate matter but may result in increased noise, which can disrupt the teaching environment. We selected an intensity level that complies with the recommended limit of 35 dB(A). While some studies suggest that sound levels up to 40 dB(A) are acceptable in schools [6], others have found that levels around 48 dB(A) were bothersome for several teachers, although individual tolerance varied widely [9]. In our study, the actual measured sound levels did not exceed 40 dB(A) for those closest to the purifiers. However, neither teachers nor students reported that the sound was disruptive, although some did notice it. It is also worth noting that the teachers expressed a willingness to tolerate higher noise levels if they believed the purifiers protected them and their students from infections. This suggests that the perceived benefits of air purification might justify higher noise levels, depending on the context.

This pilot used air quality measurements to study the effect of air purifiers on reductions in particulate matter, including infectious aerosols. This reduction is not necessarily equal to the decrease in exposure to microbes causing respiratory infections. When planning a larger study, selecting a valid outcome to assess the impact of air purifiers on disease transmission is crucial. Ideally, the number of confirmed infections for respiratory diseases among students should serve as the primary outcome. However, our findings suggest that obtaining sufficient consent for participation is unlikely, and incorporating self-testing for respiratory infections could further reduce response rates. This concern is supported by other studies indicating that securing consent from parents or guardians often results in lower response rates [42, 43]. An alternative is collecting routinely gathered absence data from the municipality records, which proved feasible in our study. However, inconsistencies in how teachers recorded these data affected their accuracy. Nonetheless, using student absenteeism as a proxy for respiratory disease, collected from anonymous registry data, appears to be the most feasible approach. The proposed establishment of a national absence data registry for schools in Norway [44] would be invaluable in this context; potentially providing comprehensive and reliable data that could serve as a key endpoint in larger studies on the effectiveness of air purifiers. We have not explored registry data from visits to primary health care services as an outcome in this study.

An alternative outcome could be the viral load measurements in environments with and without air purifiers, collected through aerosol samples. This was outside the scope of this pilot but has been performed in other studies [45]. Future research could potentially combine the collection of absence data and viral load measurements to provide a more comprehensive and accurate assessment of the purifiers’ effectiveness in reducing airborne disease transmission.

While both ceiling-mounted and portable air purifiers were feasible and acceptable in this study, each type presents distinct benefits and challenges if deployed in a large-scale study. Ceiling-mounted purifiers are unobstructive, minimizing the risk of tampering or being moved by students or staff. However, they need to be installed by a professional, posing logistical challenges in large-scale studies involving numerous schools or during emergencies like pandemics. Portable purifiers, on the other hand, were easy to install and can be managed by school personnel, making them more adaptable to widespread use. However, their placement sometimes interferes with classroom layout, they can be accidentally unplugged, and objects can be inserted into the units. Addressing these challenges—such as providing sufficient outlets and ensuring secure placement—is essential for optimizing the performance and safety of air purifiers in classrooms. It will also be essential to monitor air purifier functionality and adherence in future studies. Our findings indicate that manual timers and mobile app monitoring could be practical tools; however, challenges such as user errors, WiFi compatibility issues, and power interruptions should be expected.

If air purifiers are found effective in reducing disease transmissions in a larger trial, and are adopted or rolled-out universally, other costs and logistical challenges regarding classroom layout could incur. Such costs were out of the scope of this study.

Mean CO_2_ levels are often used to assess ventilation and airborne transmission risk [46]. While WHO recommends levels below 1000 ppm [47], some experts suggest a 700 ppm threshold to minimize airborne transmission risk [48]. In our study, mean CO_2_ levels in primary school classrooms were below 1000 ppm, but students experienced stuffy air and related headaches and we observed fluctuations between 700 and 800 ppm. This indicates room for air quality improvement and suggests that air purifiers could reduce transmission risk in schools.

### Strengths and limitations

A strength of our study was the diverse use of methods, including quantitative and qualitative. We carried out surveys with teachers and students, statistical analysis of effectiveness, collected registry data, and held group and individual interviews with students and teachers. Two different sensors were used to collect air quality, placed at different locations in the classrooms. We chose a three-arm randomized multiple crossover design to assess the superiority of air purifiers in part 2, to account for the daily and weekly variations in particulate matter and potential differences between classroom use.

The study also has limitations. It was conducted in a limited number of classrooms over a relatively short timeframe, which may affect the generalizability of our findings regarding effectiveness, feasibility, and acceptability. As such, it is still unclear how air purifiers in schools will perform over time in terms of filter replacement and acceptance. The group and individual interviews were conducted only with a small sample of the teachers and students in each classroom. The long-term feasibility of operating air purifiers in classrooms, particularly concerning maintenance and sustained functionality, remains uncertain. The beneficial effects on air quality and headache frequency reported by teachers and students should be interpreted with caution, as it was not part of the study’s objectives and could potentially be attributed to a placebo effect.

The effectiveness of air purifiers may vary according to classroom size and unit placement. Therefore, future studies and practical implementations should consider these variables, along with budget constraints and the noise-effectiveness ratio of different purifiers, to determine the most suitable solutions for different school environments. Although the air quality in the classrooms where efficacy was evaluated was moderate, as indicated by CO_2_ and VOC levels, the level of particulate matter was substantially lower than anticipated in our sample size calculation. This may explain the lack of a statistically significant effect from the ceiling-mounted purifiers.

## Conclusion

Overall, our findings suggest that installing and operating portable and ceiling-mounted air purifiers in classrooms is feasible and well-accepted by teachers and students. Collecting outcome data from students by acquiring consent from students’ legal guardians proved challenging due to low consent rates. Using already collected registry data on absences may overcome this challenge in a larger trial. Portable units offer an easy-to-install solution for effectively reducing particulate matter and might be superior to ceiling-mounted purifiers in classrooms. These insights are important for guiding the design and implementation of air purifier interventions in schools, both in the context of larger research studies and during pandemics.

## Supplementary Information

Supplementary Material 1.

## Data Availability

Data and analysis code are available at Zenodo (https://doi.org/10.5281/zenodo.13753352).
