# Supplementary material for: Air purifiers in classrooms for infection control: a pilot study including a randomized multiple crossover trial to evaluate effectiveness in reducing particulate matter
Source: Trials. 2026 Jul 11;27:594. doi: 10.1186/s13063-026-09839-5 (PMC13640335; doi:10.1186/s13063-026-09839-5)
Supplement: Supplementary file 1 [file 13063_2026_9839_MOESM1_ESM.pdf]

Online supplementary materials for "Air purifiers in classrooms for infection control: a pilot study including a randomized multiple crossover trial to evaluate effectiveness in reducing particulate matter"

I. Figures 2
II. Tables 5
III. Additional findings regarding the implementation and monitoring of air purifiers in classrooms as well as the feasibility of viral monitoring 13
IV. Teachers interview guide 15
V. Students interview guide 19
VI. Risk-of-bias assessment 23

I. Figures

Supplementary figure 1. Room area plans, air purifier and air quality sensor placement in school F (part 2), as well as existing supply and extract air ventilation.

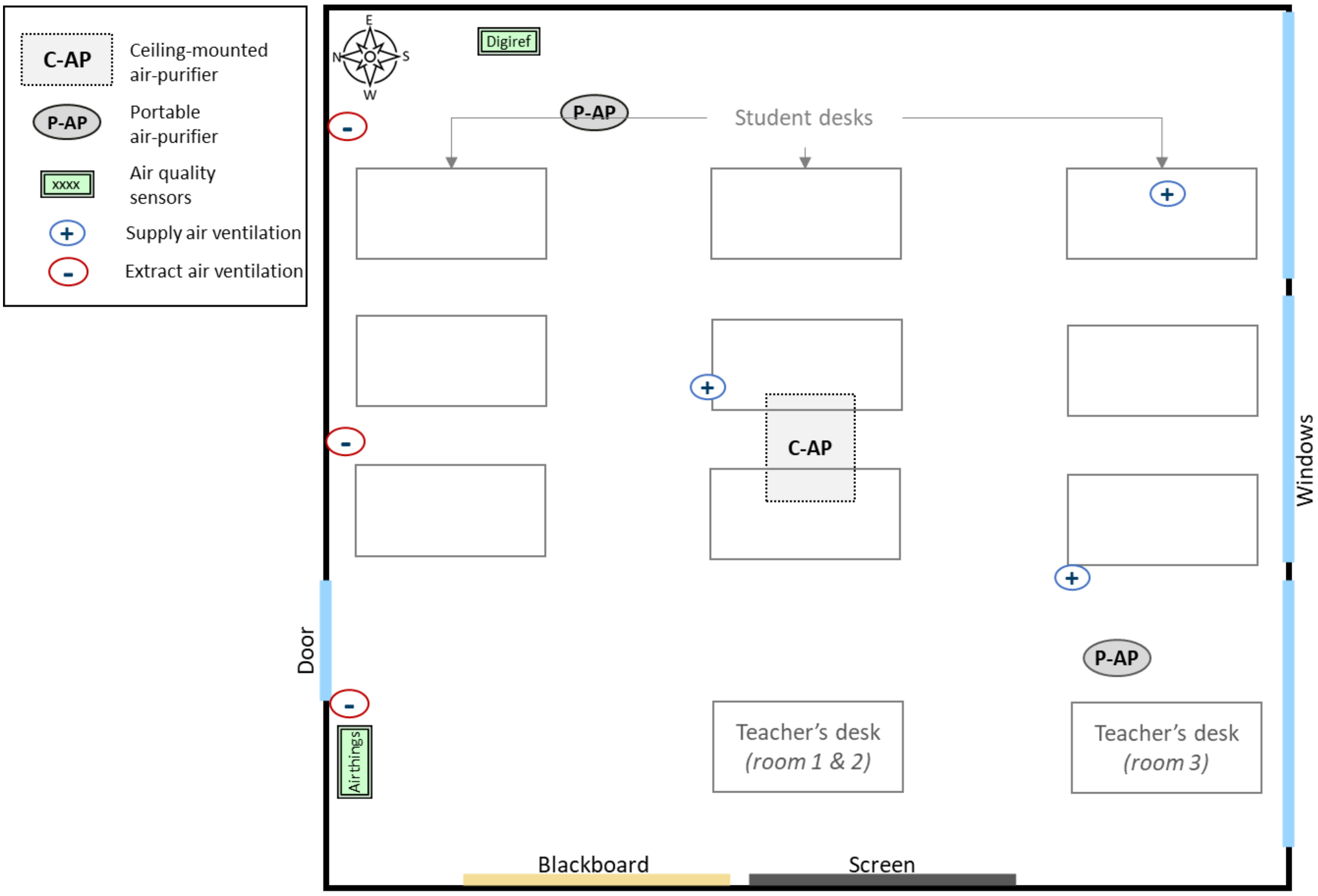

Supplementary figure 2: Weekly rate of questionnaire response among students/legal guardians, with 95% confidence intervals

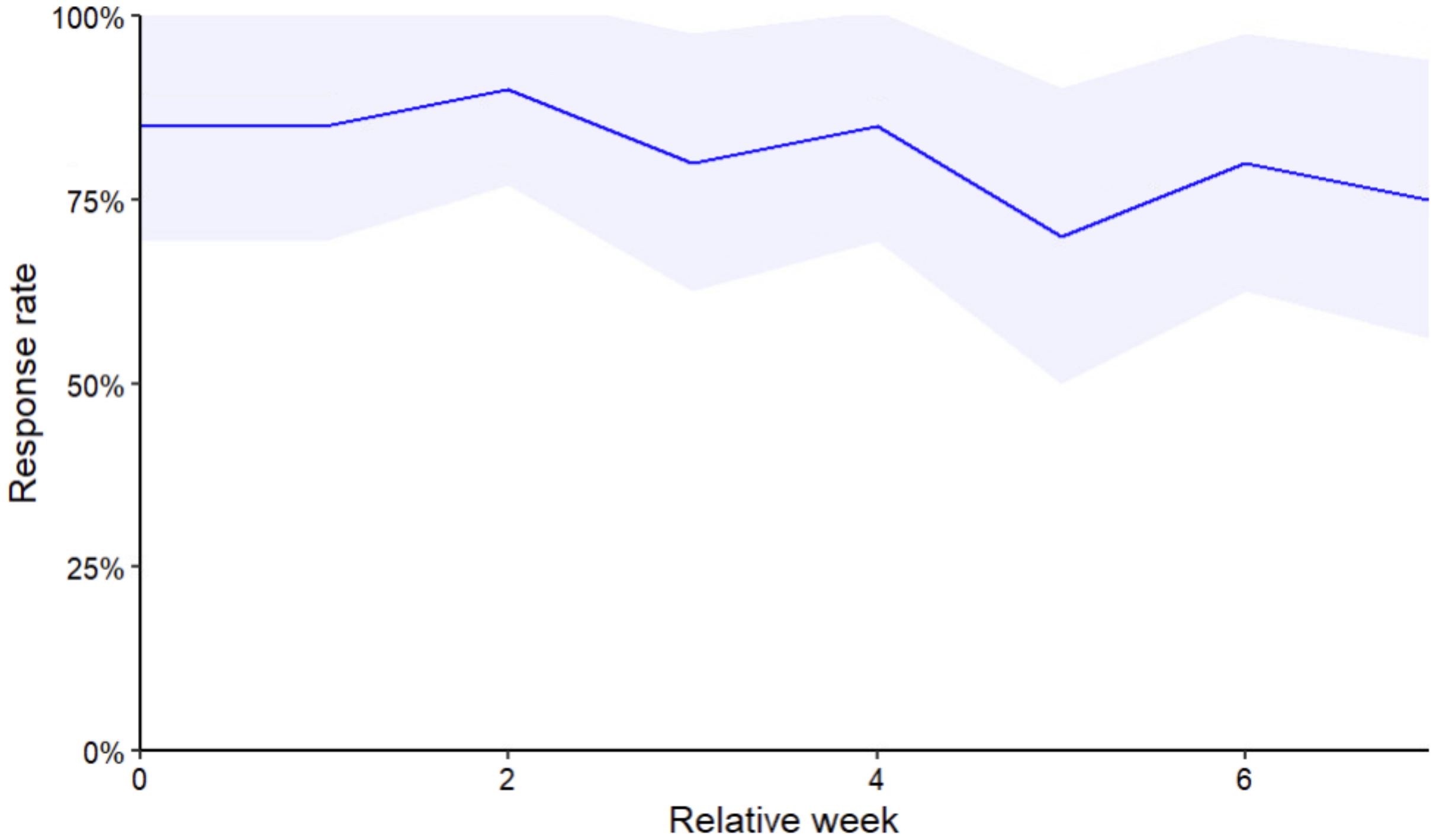

Supplementary figure 3. Mean CO_2, VOC, relative humidity, and $PM_{2.5}$ during school hours, per individual school A-E from part 1 of the study. The data covers the days when purifiers were running (all weekdays except Tuesdays) during the six-week study period. Dotted lines indicate recommended upper limit for CO_2 and recommended lower limits for humidity. The data sources of all measurements are both the Airthings and Digiref sensors, except for VOCs which is based on Airthings, as values from Digiref are not calibrated to a standard. All classes had one 15-minute break sometime between 9:30 and 10:15, as well as one 30–45-minute break between 11:15 – 12:35, during which the classrooms were unoccupied. Abbreviations: VOC, Volatile Organic Compounds; PM, Particulate matter; RH, Relative Humidity.

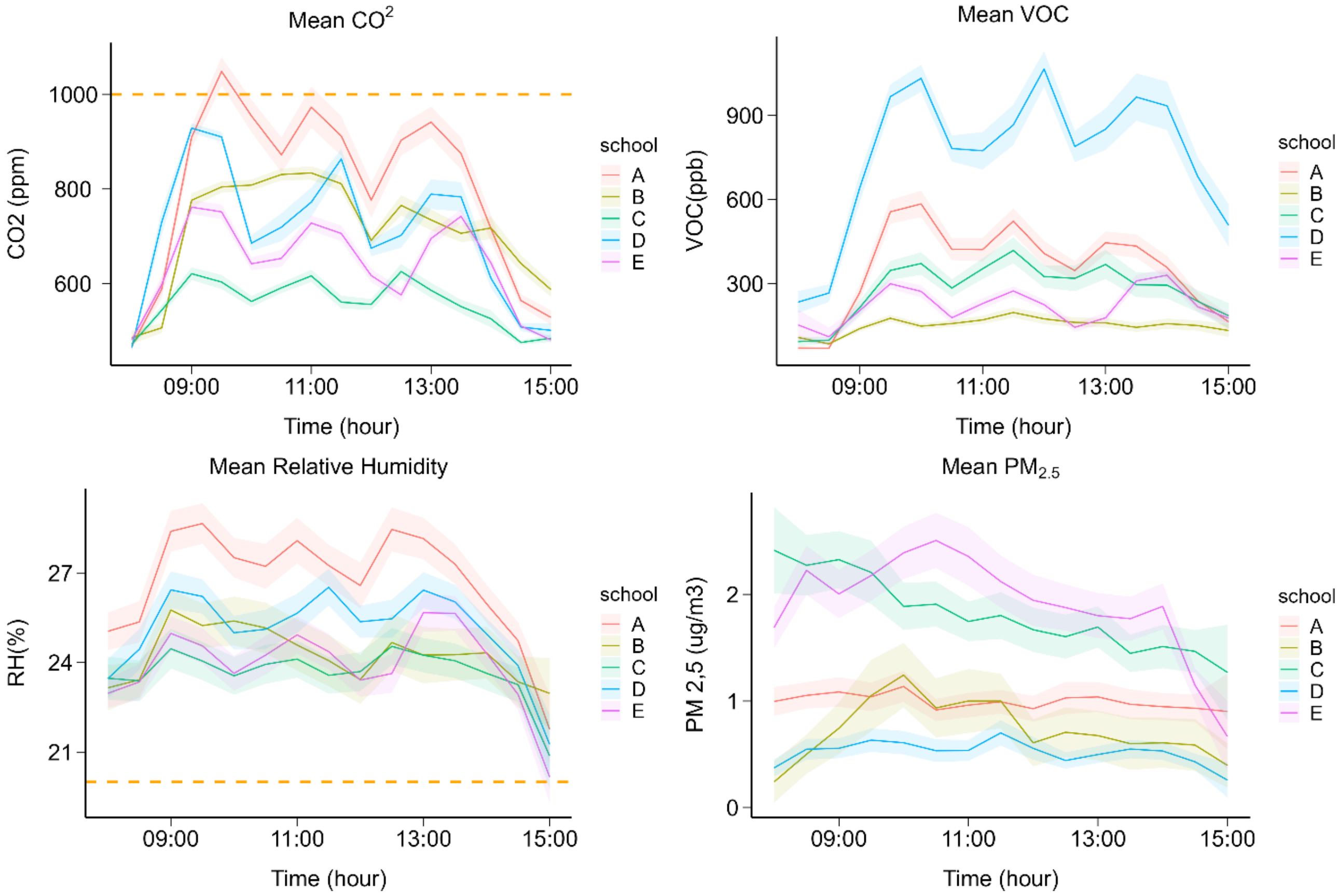

II. Tables

Supplementary table 1: CONSORT 2010 checklist of information to include when reporting a pilot or feasibility trial

Section/Topic	Item No	Checklist item	Reported on page No
**Title and abstract**			
	1a	Identification as a pilot or feasibility randomised trial in the title	1
	1b	Structured summary of pilot trial design, methods, results, and conclusions (for specific guidance see CONSORT abstract extension for pilot trials)	2-3
**Introduction**			
Background and objectives	2a	Scientific background and explanation of rationale for future definitive trial, and reasons for randomised pilot trial	5
	2b	Specific objectives or research questions for pilot trial	5
**Methods**			
Trial design	3a	Description of pilot trial design (such as parallel, factorial) including allocation ratio	8-9
	3b	Important changes to methods after pilot trial commencement (such as eligibility criteria), with reasons	15
Participants	4a	Eligibility criteria for participants	9
	4b	Settings and locations where the data were collected	9
	4c	How participants were identified and consented	9
Interventions	5	The interventions for each group with sufficient details to allow replication, including how and when they were actually administered	9-10

Outcomes	6a	Completely defined prespecified assessments or measurements to address each pilot trial objective specified in 2b, including how and when they were assessed	11, 12, 13
	6b	Any changes to pilot trial assessments or measurements after the pilot trial commenced, with reasons	13
	6c	If applicable, prespecified criteria used to judge whether, or how, to proceed with future definitive trial	NA
Sample size	7a	Rationale for numbers in the pilot trial	13
	7b	When applicable, explanation of any interim analyses and stopping guidelines	NA
Randomisation:			
Sequence generation	8a	Method used to generate the random allocation sequence	7
	8b	Type of randomisation(s); details of any restriction (such as blocking and block size)	7
Allocation concealment mechanism	9	Mechanism used to implement the random allocation sequence (such as sequentially numbered containers), describing any steps taken to conceal the sequence until interventions were assigned	7
Implementation	10	Who generated the random allocation sequence, who enrolled participants, and who assigned participants to interventions	7
Blinding	11a	If done, who was blinded after assignment to interventions (for example, participants, care providers, those assessing outcomes) and how	In SAP https://doi.org/10.5281/zenodo.12818265
	11b	If relevant, description of the similarity of interventions	NA
Statistical methods	12	Methods used to address each pilot trial objective whether qualitative or quantitative	13-15 and SAP

**Results**			
Participant flow (a diagram is strongly recommended)	13a	For each group, the numbers of participants who were approached and/or assessed for eligibility, randomly assigned, received intended treatment, and were assessed for each objective	21 and Figure 2
	13b	For each group, losses and exclusions after randomisation, together with reasons	21 and Figure 2
Recruitment	14a	Dates defining the periods of recruitment and follow-up	Table 2
	14b	Why the pilot trial ended or was stopped	NA
Baseline data	15	A table showing baseline demographic and clinical characteristics for each group	Supplementary table 2
Numbers analysed	16	For each objective, number of participants (denominator) included in each analysis. If relevant, these numbers should be by randomised group	Figure 2
Outcomes and estimation	17	For each objective, results including expressions of uncertainty (such as 95% confidence interval) for any estimates. If relevant, these results should be by randomised group	21-22
Ancillary analyses	18	Results of any other analyses performed that could be used to inform the future definitive trial	21-22
Harms	19	All important harms or unintended effects in each group (for specific guidance see CONSORT for harms)	23
	19a	If relevant, other important unintended consequences	NA
**Discussion**			
Limitations	20	Pilot trial limitations, addressing sources of potential bias and remaining uncertainty about feasibility	26-27 and risk of bias assessment

Generalisability	21	Generalisability (applicability) of pilot trial methods and findings to future definitive trial and other studies	24-25
Interpretation	22	Interpretation consistent with pilot trial objectives and findings, balancing potential benefits and harms, and<br><br>considering other relevant evidence	24-25
	22a	Implications for progression from pilot to future definitive trial, including any proposed amendments	24-25
**Other information**			
Registration	23	Registration number for pilot trial and name of trial registry	7
Protocol	24	Where the pilot trial protocol can be accessed, if available	7
Funding	25	Sources of funding and other support (such as supply of drugs), role of funders	28
	26	Ethical approval or approval by research review committee, confirmed with reference number	28

Supplementary table 2: Measured and declared specifications of the air purifiers used in the study.

	**Portable purifier**	**Ceiling-mounted purifier**
**From the manufacturer**		
Dimensions (mm) (w*h*d)	332 * 606 * 341	630 x 290 x1050
Weight	8.0 kg	32.0 kg
Filter	HEPA 13 + prefilter + activated carbon filter	HEPA 13 + prefilter + activated carbon filter
Sound pressure level (normal mode) (dB(A))	18.5-46	46 – 68.5
Room size (max)	115 m^2 floor area	274 m^3 vol
CADR, range	120 - 448 m^3/h	140 – 1440 m^3/h
Total CADR, at selected level	245 x 2 = 490	566
Air inlet (height)	Lower half, two sided	Bottom
Air outlet	Top	
Outlet direction	Upward	Horizontal (4 directions)
**Measured in situ**		
Sound pressure level dB(A), (Room average / most affected)		
Level 3 (Preset) level[1]	NA	34.8-36.9 / 40.5-40.6
Level 4[2]	29.8-31.1 / 30.8-33.2	NA
Level 5[3]	37.7-38.0 / 41.2-42,7	NA

Abbreviations; mm; millimeter, w, width; h, height; d, depth; CADR, clean air delivery rate, dB(A); decibels A weighted.

[1]The level used for the ceiling-mounted purifiers during the study period.

[2]The level used for the portable purifiers during the study period.

[3]The level tested after the study period in selected schools.

Supplementary table 3: Characteristics of schools and classrooms that participated in the study.

School	Class-room	Built/ Last renovated	Classroom volume (m^3)	Ceiling height (m)	Openable windows (no/area (m^2))	Floor area (m^2)	Existing ventilation type	Declared no of students[1]	Orientation of classroom (Direction - View)	Surroundings	Air purifier type[2]
Part 1											
A	1	1912/2011	195.7	3.53	6/4[3]	55.4	Balanced mechanical	20	South – Schoolyard	Urban	Portable
B	1	2020/ -	196.9	2.91	2/2.1	67.7	Balanced mechanical	22	East – Sports field	Suburban	Portable
C	1	1924/2008	191.6	2.80	6/8[3]	68.4	Balanced mechanical	27	West – Schoolyard	Suburban	Portable
D	1	1934 / 2009	155.1	2.81	9/12[3]	55.2	Balanced mechanical	20	South – Schoolyard	Suburban	Ceiling-mounted
E	1	1957/ -	171.9	2.90	2/2.9[3]	59.3	Balanced mechanical	25	South – Schoolyard	Suburban	Ceiling-mounted
Part 2											
F	1	1958	170.1	2.87	2/2.9	59.3	Balanced mechanical	8 (M/W/F), 20 (Tu/Th)	South – Schoolyard	Rural	Both types
F	2	1958	170.1	2.87	2/2.9	59.3	Balanced mechanical	7 (M/W/F), 20 (Tu/Th)	South – Schoolyard	Rural	Both types
F	3	1058	170.1	2.87	2/2.9	59.3	Balanced mechanical	16 (M/W/F), 8 (Tu/Th)	South – Schoolyard	Rural	Both types

[1] Number of students in the class using the classroom.

[2] Classrooms with portable purifiers had two devices, while classrooms with ceiling-mounted purifiers had one device.

[3] Area approximated from photo.

Supplementary table 4: Baseline summary statistics for air quality parameters in each school in part 1 and 2 of the study, the week before starting the intervention, during teaching hours. The numbers are based on measurements from both Airthings and Digiref sensors.

	Part 1					Part 2		
	**School A**	**School B**[1]	**School C**	**School D**	**School E**	**School F**		
**Classroom**	**1**	**1**	**1**	**1**	**1**	**1**	**2**	**3**[2]
Temperature, °C								
Median	20.9	NA	21.2	21	22.6	22.8	24.2	22.6
Avg (SD)	20.9 (1.8)	NA	21.3 (0.5)	21.1 (0.7)	22.4 (0.9)	23.1 (1.4)	24 (1.4)	22.4 (1.4)
Range (min-max)	14.6 - 24.2	NA	20 - 22.7	19.8 - 22.6	21.1 - 24	17.9 - 26.1	20.8 - 29.2	19.1 - 25.8
RH, %								
Median	19	NA	16	19	18.4	23.9	19.5	23.1
Avg (SD)	19 (5.2)	NA	15.4 (4.6)	18.6 (4)	18 (4.1)	23.4 (4.6)	19.5 (1.9)	23 (4.3)
Range (min-max)	7.7 - 30	NA	6.2 - 23	8.7 - 28.1	9.2 - 26	12.3 - 34	15.7 - 24.6	12.2 - 33
VOC, ppb								
Median	370	NA	202	448	186.5	163	195	256.5
Avg (SD)	426.7 (309.7)	NA	254.3 (213.7)	576.4 (382.4)	223.8 (177.9)	206.8 (183.1)	241.6 (152.7)	256.6 (150.1)
Range (min-max)	15 - 2230	NA	12 - 957	46 - 1849	4 - 619	1 - 864	46 - 574	8 - 690
CO_2, ppm								
Median	849.5	NA	539	764	724	1128.5	1046	1121
Avg (SD)	804 (248.3)	NA	562.7 (101)	736 (186.3)	694.8 (168.2)	1182.4 (444.1)	1018.9 (316.9)	1104.1 (399.9)
Range (min-max)	395 - 1268	NA	395 - 869	415 - 1091	398 - 1151	398 - 2376	429 - 1764	410 - 1926
$PM_{2.5}$, µg/m^3								
Median	3.2	NA	4.4	0.6	5.7	1	1	1
Avg (SD)	3.4 (2.1)	NA	4.3 (2.7)	0.8 (1)	5.6 (3.4)	2.1 (2.7)	1.9 (2.4)	1.4 (1.5)
Range (min-max)	0 - 11	NA	0 - 14	0 - 7	0 - 19	0 - 20	0 - 12	0 - 9

Abbreviations; SD, Standard Deviation; min-max, the minimal and maximal measured absolute values; RH, Relative Humidity; VOC, Volatile Organic Compounds; NA, Not Available. [1]No baseline measurements were available from school B as the Digiref sensor was defective and the Airthings sensor was accidentally unplugged. Both were up and running as of study week 1. [2]The CO_2 measurements from classroom 3, School F, are only based on the Digiref sensor, as the Airthings sensor did not register CO_2 in this classroom due to an error.

Supplementary table 5: Exploratory time-varying covariate adjusted analysis. The time-varying covariates selected by lasso were indoor relative humidity, weekday, baseline morning Particulate Matter ($PM_{2.5}$), and the setting of the school's existing ventilation system setting.

	None	Ceiling	Portable	Ceiling	Portable	Superiority of air purification[3]	Noninferiority (portable vs ceiling)[4]
	Mean[1]	Mean[1]	Mean[1]	RR[2] (95% CI)	RR[2] (95% CI)		
$PM_{2.5}$	1.03	0.84	0.55	0.93 (0.77-1.12)	0.71 (0.64-0.78)	<0.001	<0.001

Abbreviations: PM, Particulate Matter; VOC, Volatile Organic Compounds

[1]Sample means are unadjusted and do no account for the crossover design.

[2]Rate ratios (RRs) are adjusted for the crossover design, sensor type, first-order autocorrelation, the time-varying covariates selected by the cross-validated lasso, and clustering within classroom. RR < 1 favors either ceiling-mounted or portable air purifiers over the reference (no air purification).

[3]Two-sided p-values for the joint null hypothesis $H_0: \log \mathrm{RR}_{\mathrm{CN}} = \log \mathrm{RR}_{\mathrm{PN}} = 0$, where $\mathrm{RR}_{\mathrm{CN}}$ and $\mathrm{RR}_{\mathrm{PN}}$ are rate ratios comparing ceiling-mounted and portable air purifiers to no air purifiers, respectively.

[4]One-sided p-values for the null hypothesis $H_0: \log \mathrm{RR}_{\mathrm{PC}} \geq \log m$, where $\mathrm{RR}_{\mathrm{PC}}$ is a rate ratio comparing portable to ceiling-mounted air purifiers and $\log m$ is the prespecified noninferiority margin.

III. Additional findings regarding the implementation and monitoring of air purifiers in classrooms as well as the feasibility of viral monitoring

Operating the air purifiers

To ensure a smoother implementation of air purifiers in a larger study, we investigated various methods of operating the devices, with the goal of reducing the workload on teachers. From our evaluation, using a manual timer appeared as the most suitable option to test in the pilot study. The timer offered several including its resilience to power interruptions due to its internal battery, which lasted for xx hours. Additionally, it effectively turned the devices on and off according to the predetermined schedule. However, there were some disadvantages, including its relatively large size, blocking both sockets when plugged in. Further, even though the device setup was not overly complex, there was a significant chance of user errors when configuring the timers, in particular if different settings were required at different days. We identified at least one such case where the time schedule was not correctly specified. Moreover, using a timer assumes that the purifier will automatically start at the predetermined level after being turned off and on by the timer, which is not the case for all portable purifiers.

Monitoring the air purifiers

In the context of monitoring the functionality and adherence to the planned schedule of the air purifiers in a larger study, we explored a Bluetooth smart contact connected to an app. Additionally, we tested the Mill app from Mill, which relied on a Wi-Fi connection. Unfortunately, the school's Wi-Fi operated on the 5Ghz frequency and was thus incompatible with the Mill portable purifier. As an alternative solution, we tested a portable Wi-Fi hotspot in school F (part 2) to connect all six portable purifiers from all classrooms to the app. Monitoring through this app was successful, allowing us to easily evaluate whether the individual purifiers were operating as intended. However, we encountered a challenge with the WiFi-device, as it was consistently turned off whenever there was a

power outage, requiring manual reactivation. Using the Bluetooth smart contact, we could schedule when the air purifying devices had access to electricity. The configuring of timers was slightly more user friendly that with the manual timers. This scheduling could only occur when the phone with the app was in Bluetooth connection with the smart contact, thus long-distance surveillance was impractical. Furthermore, if the smart contact was withdrawn from the socket or there was a power outage, the settings were lost.

Feasibility of viral monitoring

Monitoring the presence of viruses in the study classrooms may of relevance in a larger study, to see what types of viruses that are circulating and potentially causing disease and absence. We initially planned to test for viruses on regular swipes of the HEPA-filters on the portable purifiers. However, the Mill purifier has a combined prefilter and HEPA-filter, thus sampling of the HEPA-filter without destroying the combined filter is not feasible. We also considered using aerosol-samplers, however, the cost for samplers with sufficiently low sound levels were beyond the budget for the present pilot study. We did not attempt to sample the ceiling-mounted filter.

IV. Teachers interview guide

Group interview with teachers – EXPLORATION OF ACCEPTABILITY (Translated from Norwegian)

Conducted during the first couple of weeks and during the final week of the intervention, in part 1 and part 2 of the pilot study.

**Date:**	
**Interview nr:**	
**Leader / Observer(s)**	
**Audio / Screen recording?**	

BEFORE THE DISCUSSION: INTRODUCTION AND CONSENT Thank you very much for taking the time to participate in this interview, we truly appreciate it.

Presentation of participants and researchers

- **Participants:** Name, workplace?
- **Researchers:** Name, position, and background (we are not experts – we are here to learn).

Inform participants about their rights and our responsibilities

You have consented to us making a recording, and I will briefly explain your rights and our responsibilities:

All data we collect will be treated confidentially. Your name and contact information will not be linked to the recording or the interview notes, nor stored together with them. The recording will be stored on a password-protected server. It will be transcribed, but no names will be used in the transcription files. Only researchers from the data collecting institution will have access to the recording files. The recording will be deleted from the secure server after transcription, and no later than two months afterwards.

You have the right to withdraw from participation at any time, and you may also request that your information and the recording be deleted after the interview. In this focus group, we will not collect health information. Therefore, it is important that you do not disclose anything about your own health, or the health of colleagues or people you know.

Briefly about the Air Purifier Study and why we are conducting this pilot study

We are carrying out interviews/group discussions to gain deeper insight into the classroom context and to explore your perspectives and experiences. It is important for us to hear how it has been so far to take part in the study, for example regarding communication and workload related to it. In addition, we would like to hear about your experiences with the air purifiers and how it has worked in your daily routines to have them in your workplace. This is important information for us, which we cannot obtain in any other way. We would like you to tell us both the positive and negative aspects of the project, to be honest, and to share your experiences.

We have asked you to join us because you are the ones who know what happens in the classroom. You can give us valuable information that we can use when planning the larger study. Today, it is us who will be

learning from you! You can tell us things we did not know about what it is like to have air purifiers in the classroom. And that is what we hope to hear from you.

What we are doing here today is not a test, and there are no right or wrong answers to the questions we ask. So here you can say whatever you are thinking.

It is helpful for us if you comment on what the others in this discussion say – we are curious to know whether you agree or disagree with each other, and if you want to elaborate on something for us, that is great.

Do you have any questions before we start the recording?

Group interview begins / Recording starts

Theme 1: Studies in schools (approx. 5 minutes) (only part 1 groups)

What kinds of experiences have you had with studies conducted in schools?

- Prompts: Do they feel time-consuming? Exciting? Meaningful?
- Teachers' contributions are important in school studies – but we know that teachers have a busy everyday life. What are good ways to involve teachers, balancing participation with time demands?
- What is needed for a study to feel useful and not just burdensome?
- How has it been to take part in this study so far?

(Transition) To go a bit more into detail about this study, we would like to ask:

Theme 2: Information (approx. 10 minutes) (only part 1 groups)
How have you experienced the information provided by us in this study?

- Do you think the information has been useful?
- o Too little, too much?
- Was anything unclear or difficult to understand?
- How has the process of collecting consent from parents/guardians worked?
- o What do you think about school messaging vs. paper forms?
- Have there been any questions from parents or students about this / about the information?
- o If yes, can you give some examples?
- o Has it created a lot of extra work for you?
- How would you prefer that we collect consents in another study?

Theme 3: Air quality in the classroom (approx. 5 minutes)

How do you think the air usually is in the classroom?

- How often do you usually air out the classroom, and who is responsible for this?
- Have you ever thought that the air in a classroom was good or bad?
  - If yes, what do you think is the reason that the air is good/bad?
  - Is there a difference in how the air feels in the different classrooms? If yes, in what way?
  - Does the air change during the course of the school day?

Theme 4: Experience of air purifiers (approx. 15-20 minutes)
What is it like to have the air purifiers in the classroom?

- How do you feel about having an air purifier in the classroom? Do you think about it at all?
- In what ways do you notice that there have been air purifiers in the classroom?
- To what extent have you noticed any difference in the air?
- Do you have any advice or suggestions on what you think would be the best way to use air purifiers in the classroom?
- What do you think about us asking you to monitor whether the air purifiers are on or off?
- What do you think about us asking you to turn the air purifiers on and off every day?
  - Responsibility? Time? Workload?
- Have there been any complaints from the students:
  - Noise?
  - Temperature?
  - Draft?
  - Dry air?
  - Bad smell?
  - Dust in the air?

Theme 5: Virus testing (max 10 minutes) (only part 1 groups)

If we were to conduct such a study on a larger scale, we might want to know whether participants – both teachers and students – contracted COVID-19 or other similar illnesses during the study period. What are your immediate thoughts about this?

- What would you think if we asked teachers to test themselves regularly?
  - When experiencing symptoms?

Member checking, summary **(only part 1 groups)**:

- Do you find the air purifiers disruptive?
- Do you find that the presence of the air quality monitors in any way interferes with teaching?

- Do you find the air purifiers bothersome?
- Do you find the air purifiers bothersome for the students?

Additional comments

- Is there anything else you would like to add?
- What do you think about the feasibility of conducting such a study on a much larger scale?
- How do you think schools can be engaged and brought on board?
- How was it to take part in this interview? Would you consider participating again? Would you consider having another conversation with us after six weeks?

If you would like, we can explain a bit more about why we are conducting this pilot, what the purpose of the larger study will be, how we plan to carry it out, and how the effects of the air purifiers can be measured.

Thank the participants for their time and insights.

Only for part 2 interview:

Theme 3: Having air purifiers permanently / during a pandemic (approx. 5 minutes)

Based on your experiences so far – what do you think about having air purifiers in the classroom:

- Permanently?
  - If no – what if we can show that they have an effect on absenteeism?
- During a pandemic?
  - Why / why not?
- If you had air purifiers in the classroom as part of a study over a longer period – how would it be for you to monitor whether they were on or off?

V. Students interview guide

Group interview with students – EXPLORATION OF ACCEPTABILITY (Translated from Norwegian)

Conducted at the end of the intervention, in part 1 of the pilot study.

**Date:**	
**Interview nr:**	
**Leader / Observer(s)**	
**Audio / Screen recording?**	

Prior to the discussion: Introduction and informed consent.

Important: Parental consent must be obtained beforehand.

Present yourself and explain your role in the study. Collect the children's names and ages.

Intro

Brief introduction about this research project: We want to study whether air quality improves when air purifiers are placed in classrooms. Before starting a large-scale study, we are conducting a smaller pilot study to help us understand whether this approach is feasible. We have invited you to take part because you are actually the experts on what happens in the classroom. We know nothing about that! You can provide us with truly valuable information that we can use in the larger study. You may tell us things we absolutely did not know about what it is like to have air purifiers in the classroom.

Today, it is us who will be learning from you! We hope you can tell us as much as possible about what you think and feel. What you say is really important to us. Nothing is wrong! We might ask and dig a little, because the more you talk – the more we learn.

What we are doing here today is not a test, and there are no right or wrong answers to the questions we ask. So here you can really say whatever comes to mind.

It is helpful for us if you comment on what the others in this discussion say – we are curious to know whether you agree or disagree with each other, and if you want to elaborate on something for us, that's great.

Before we start, I will say a few important things to those of you participating in this group discussion:

1. Participation in this conversation is completely voluntary, and what you say here will not be shared with anyone else.
2. We will record the interview, and your parents have already given consent for this.
3. In today's discussion, we will talk about the air purifiers and what it is like to have them in the classroom. We will also ask you what you think about COVID-19 testing. But there is

one important point: we do not want to know anything about your own health or illnesses, or the health of anyone you know. This means we do not want to hear stories about when you were sick, tested positive, or anything else directly related to your health. This is because we are not allowed to collect such information, and we are making a recording of what is said here today. What we want to hear are your general thoughts about testing, for example.

4. If you decide that you no longer want to participate or wish to end the conversation, you may do so at any time without giving a reason.

Do you have any questions? Group begins interview / Recording starts.

Ice-breaker – Warm-up exercise

Before we start the real conversation here, I would like you to describe your dream classroom. Tell us what it would look like if you could decide. What would you have in the classroom? And why?

After the exercise: Now we will move on to the core topics we are curious about. We will talk about three themes: the air in the classroom, air purifiers in the classroom, and what you think about testing.

Theme 1: Air quality in the classroom
How do you think the air usually is in the classroom?

- What does the classroom look like, where is it located, how many classrooms do you use during the school day, can you stay inside the classroom during recess, etc.?
- Is there a difference in how the air feels in the different classrooms?
- If yes, in what way?
- Have you ever thought that the air in a classroom was good or bad?
- If yes, what do you think is the reason that the air is good/bad?
- Does the air change during the course of the school day?

Theme 2: Experience of air purifiers
What was it like to have the air purifiers in the classroom?

- How many air purifiers do you have, where are they placed, what do they look like, etc.?
- How do you feel about having an air purifier in the classroom?
- Can you tell us a bit about what it has been like during this period?
- To what extent have you noticed any difference in the air?
- Do you have any advice or suggestions on what you think would be the best way to use air purifiers in the classroom?
- What would you think if you were told that the air purifiers would remain for the rest of the year?
- If you could choose, would you like to continue having an air purifier in the classroom, or not?
- Why / why not?

Theme 3: Virus testing

If we were to conduct such a study again, we might want to know whether you contracted COVID-19 or other similar illnesses during the study period. How would it feel for you if you had to test every time you developed symptoms?

- What do you think about testing before going to school, using a rapid test?
- What would motivate you to participate in testing in a study?
- What would make you decide not to test yourself?

Additional comments

- Is there anything else you would like to add?
- How was it to take part in this? Would you consider participating again?

If you would like, we can explain a bit about why we are conducting this pilot, what the purpose of the larger study will be, how we plan to carry it out, and how the effects of the air purifiers can be measured.

Thank the participants for their time and insights.

VI. Risk-of-bias assessment

Revised Cochrane risk-of-bias tool for randomized crossover trials

Study details

**Reference**	Air purifiers in classrooms for infection control: a pilot study including a randomized multiple crossover trial to evaluate effectiveness in reducing particulate matter. Manuscript

Study design

☐ Individually-randomized parallel-group trial
☐ Cluster-randomized parallel-group trial
X Individually randomized cross-over (or other matched) trial

For the purposes of this assessment, the interventions being compared are defined as

Experimental:	- Portable air purifiers<br>- Ceiling mounted air purifiers	Comparator:	No air purifiers

**Specify which outcome is being assessed for risk of bias**	Particulate Matter $(PM)_{2.5}$
**Specify the numerical result being assessed.** In case of multiple alternative analyses being presented, specify the numeric result (e.g. RR = 1.52 (95% CI 0.83 to 2.77) and/or a reference (e.g. to a table, figure or paragraph) that uniquely defines the result being assessed.	Portable air purifiers: Rate risk = 0.71 (95% CI 0.57 to 0.89)<br>Ceiling mounted air purifiers: Rate risk = 0.85 (95% CI 0.65 to 1.11)

Is the review team's aim for this result…?

X to assess the effect of *assignment to intervention* (the 'intention-to-treat' effect)

☐ to assess the effect of *adhering to intervention* (the ‘per-protocol’ effect)

If the aim is to assess the effect of *adhering to intervention*, select the deviations from intended intervention that should be addressed (at least one must be checked):

☐ occurrence of non-protocol interventions
☐ failures in implementing the intervention that could have affected the outcome
☐ non-adherence to their assigned intervention by trial participants

Which of the following sources were obtained to help inform the risk-of-bias assessment? (tick as many as apply)

X Journal article(s) with results of the trial
☐ Trial protocol
☐ Statistical analysis plan (SAP)
☐ Non-commercial trial registry record (e.g. ClinicalTrials.gov record)
☐ Company-owned trial registry record (e.g. GSK Clinical Study Register record)
☐ “Grey literature” (e.g. unpublished thesis)
☐ Conference abstract(s) about the trial
☐ Regulatory document (e.g. Clinical Study Report, Drug Approval Package)
☐ Research ethics application
☐ Grant database summary (e.g. NIH RePORTER or Research Councils UK Gateway to Research)
☐ Personal communication with trialist
☐ Personal communication with the sponsor

Risk of bias assessment

Responses underlined in green are potential markers for low risk of bias, and responses in red are potential markers for a risk of bias. Where questions relate only to sign posts to other questions, no formatting is used.

Domain 1a: Risk of bias arising from the randomization process

Signalling questions	Comments	Response options
**1.1 Was the allocation sequence random?**	Yes, the three classrooms were randomly assigned to one allociation sequence	N
**1.2 Was the allocation sequence concealed until participants were enrolled and assigned to interventions?**	No, but it is not relevant here as the unit is classrooms and classrooms cannot change their behaviour	N
**1.3 Did baseline differences between intervention groups at the start of the first period suggest a problem with the randomization process?**	There were only three classrooms randomised, and there were some baseline differences, see Supplementary table 2 and 3	PY
**Risk-of-bias judgement**		Some concerns
Optional: What is the predicted direction of bias arising from the randomization process?	NA	NA / Favours experimental / Favours comparator / Towards null /Away from null / Unpredictable

Domain S: Risk of bias arising from period and carryover effects

Signalling questions	Comments	Response options
**S.1 Was the number of participants allocated to each of the two sequences equal or nearly equal?**	Yes	N
**S.2 If N/PN/NI to S.1: Were period effects accounted for in the analysis?**	NA	NA
**S.3 Was there sufficient time for any carryover effects to have disappeared before outcome assessment in the second period?**	Yes, there was the weekend between	**N**
**Risk-of-bias judgement**		Low
Optional: What is the predicted direction of bias arising from period and carryover effects?		NA / Favours experimental / Favours comparator / Towards null /Away from null / Unpredictable

Domain 2: Risk of bias due to deviations from the intended interventions (effect of assignment to intervention)

Signalling questions	Comments	Response options
**2.1. Were participants aware of their assigned intervention during each period of the trial?**	NA	N
**2.2. Were carers and people delivering the interventions aware of participants' assigned intervention during each period of the trial?**	The intervention was not administered by any individual directly. Instead, various methods were employed to control the portable air purifiers, including the use of electronic timers, Bluetooth-enabled smart plugs, and corresponding mobile applications. For the ceiling-mounted units, they were pre-programmed by the supplier to operate according to the predefined schedules.	N
**2.3. If Y/PY/NI to 2.1 or 2.2: Were there deviations from the intended intervention that arose because of the trial context?**	NA	NA
**2.4 If Y/PY to 2.3: Were these deviations likely to have affected the outcome?**	NA	NA
**2.5. If Y/PY/NI to 2.4: Were these deviations from intended intervention balanced between groups?**	NA	NA
**2.6 Was an appropriate analysis used to estimate the effect of assignment to intervention?**	Analysis followed the statistical analysis plan, which was based on feedback from experts. The outcomes were analysed as counts, but could potentially also be analysed as positive continuous outcomes.	PN
**2.7 If N/PN/NI to 2.6: Was there potential for a substantial impact (on the result) of the failure to analyse participants in the group to which they were randomized?**		NA
**Risk-of-bias judgement**		Low
Optional: What is the predicted direction of bias due to deviations from intended interventions?		NA / Favours experimental / Favours comparator / Towards null /Away from null / Unpredictable

Domain 2: Risk of bias due to deviations from the intended interventions (effect of adhering to intervention)

Signalling questions	Comments	Response options
**2.1. Were participants aware of their assigned intervention during each period of the trial?**	NA	N
**2.2. Were carers and people delivering the interventions aware of participants' assigned intervention during each period of the trial?**	Air purifiers cannot be aware of delivery of intervention. However, teachers and researchers turning the devices on and off were aware of participants' (classrooms) assigned intervention	PN
**2.3. [If applicable:] If Y/PY/NI to 2.1 or 2.2: Were important non-protocol interventions balanced between interventions?**		
**2.4. [If applicable:] Were there failures in implementing the intervention that could have affected the outcome?**	There were a few disruptions in operation due to technical issues	PN
**2.5. [If applicable:] Was there non-adherence to the assigned intervention regimen that could have affected participants' outcomes?**	NA	N
**2.6. If N/PN/NI to 2.3, or Y/PY/NI to 2.4 or 2.5: Was an appropriate analysis used to estimate the effect of adhering to the intervention?**	NA	NA
**Risk-of-bias judgement**		Some concerns
Optional: What is the predicted direction of bias due to deviations from intended interventions?		NA / Favours experimental / Favours comparator / Towards null /Away from null / Unpredictable

Domain 3: Risk of bias due to missing outcome data

Signalling questions	Comments	Response options
**3.1 Were data for this outcome available for all, or nearly all, participants randomized?**	Yes	N
**3.2 If N/PN/NI to 3.1: Is there evidence that the result was not biased by missing outcome data?**	There was no missing outcome data for PM 2.5	NA
**3.3 If N/PN to 3.2 Could missingness in the outcome depend on its true value?**	NA	NA
**3.4 If Y/PY/NI to 3.3: Is it likely that missingness in the outcome depended on its true value?**	NA	NA
**Risk-of-bias judgement**		Low
Optional: What is the predicted direction of bias due to missing outcome data?		NA / Favours experimental / Favours comparator / Towards null /Away from null / Unpredictable

Domain 4: Risk of bias in measurement of the outcome

Signalling questions	Comments	Response options
**4.1 Was the method of measuring the outcome inappropriate?**	The sensors captured the concentration of PM 2.5 accurately – however their placement in the classrooms may affect the interpretation of the results	PN
**4.2 Could measurement or ascertainment of the outcome have differed between interventions within each sequence?**	The sensors placement could have shifted by accident which may have affected measurements	PN
**4.3 If N/PN/NI to 4.1 and 4.2: Were outcome assessors aware of the intervention received by study participants?**	Outcome was objectively measured by devices and not affected by the assessors.	N
**4.4 If Y/PY/NI to 4.3: Could assessment of the outcome have been influenced by knowledge of intervention received?**	No	NA
**4.5 If Y/PY/NI to 4.4: Is it likely that assessment of the outcome was influenced by knowledge of intervention received?**		NA
**Risk-of-bias judgement**	Low	Low
Optional: What is the predicted direction of bias in measurement of the outcome?		NA / Favours experimental / Favours comparator / Towards null /Away from null / Unpredictable

Domain 5: Risk of bias in selection of the reported result

Signalling questions	Comments	Response options
**5.1 Were the data that produced this result analysed in accordance with a pre-specified analysis plan that was finalized before unblinded outcome data were available for analysis?**	No, the analysis plan was published before the analysis commenced but the analysis was not unblinded	PN
**Is the numerical result being assessed likely to have been selected, on the basis of the results, from...**		
**5.2. ... multiple eligible outcome measurements (e.g. scales, definitions, time points) within the outcome domain?**	No, the outcome was prespecified.	N
**5.3 ... multiple eligible analyses of the data?**	No, the analysis was prespecified.	N
**5.4 Is a result based on data from both periods sought, but unavailable on the basis of carryover having been identified?**	There were multiple periods, and all periods were included.	**N**
**Risk-of-bias judgement**		Low
Optional: What is the predicted direction of bias due to selection of the reported result?		NA / Favours experimental / Favours comparator / Towards null /Away from null / Unpredictable

Overall risk of bias

**Risk-of-bias judgement**		Low / Some concerns
Optional: What is the overall predicted direction of bias for this outcome?		NA / Favours experimental / Favours comparator / Towards null /Away from null / Unpredictable
